# Supplementary figures and images for: PCH-2 collaborates with CMT-1 to proofread meiotic homolog interactions
Source: PLoS Genet. 2020 Jul 30;16(7):e1008904. doi: 10.1371/journal.pgen.1008904 (PMC7433886; doi:10.1371/journal.pgen.1008904)

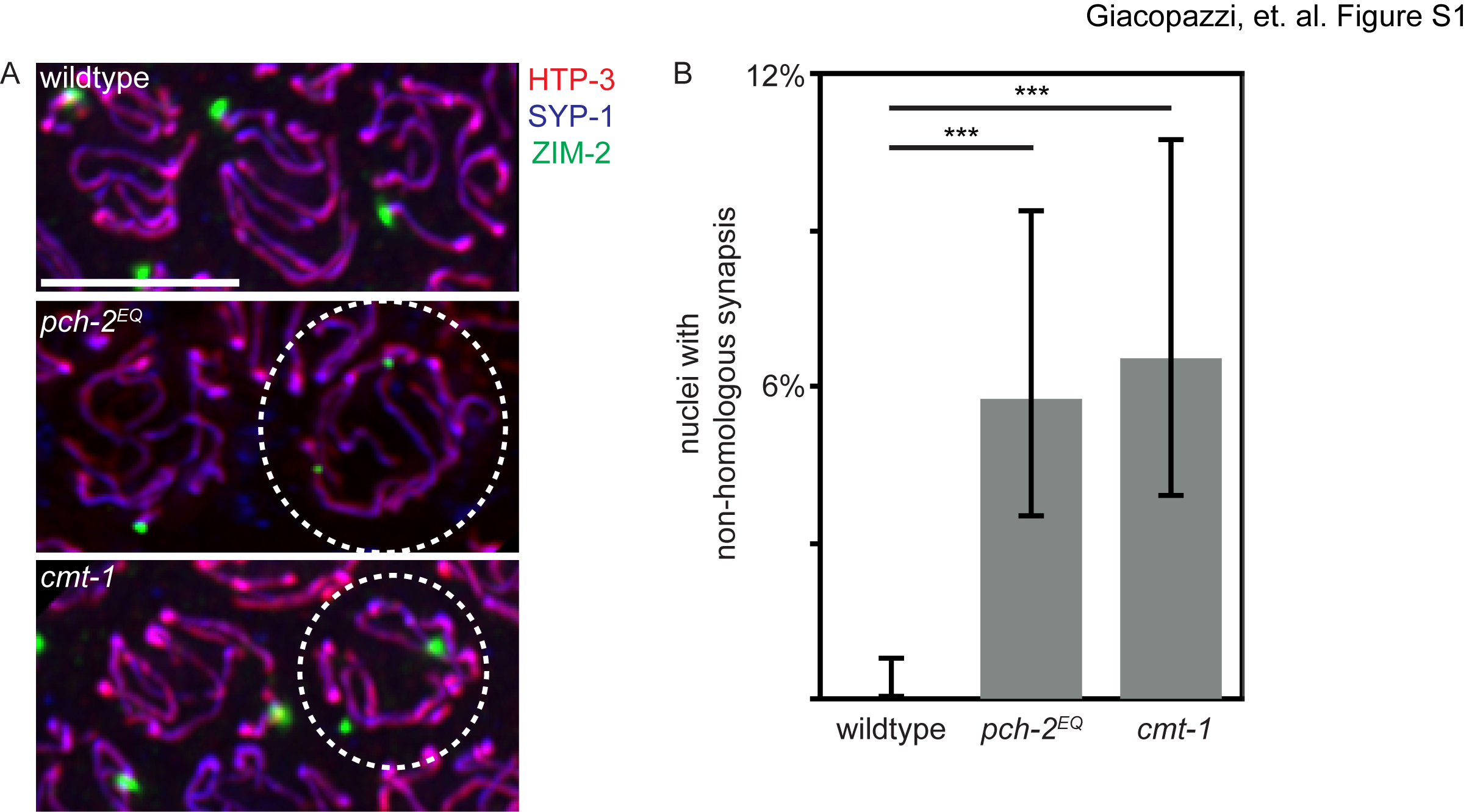

Supplement: S1 Fig — A. Images of meiotic nuclei stained with antibodies against HTP-3, SYP-1 and ZIM-2 in wildtype animals, pch-2E253Q and cmt-1 mutants. Circled nuclei have undergone non-homologous synapsis. B. Quantification of non-homologous synapsis wildtype animals, pch-2E253Q and cmt-1 mutants. Error bars indicate 95% confidence intervals. Significance was assessed by performing two-tailed Fisher exact tests. A *** indicates a p value < 0.0001. (TIF) [file pgen.1008904.s001.tif]

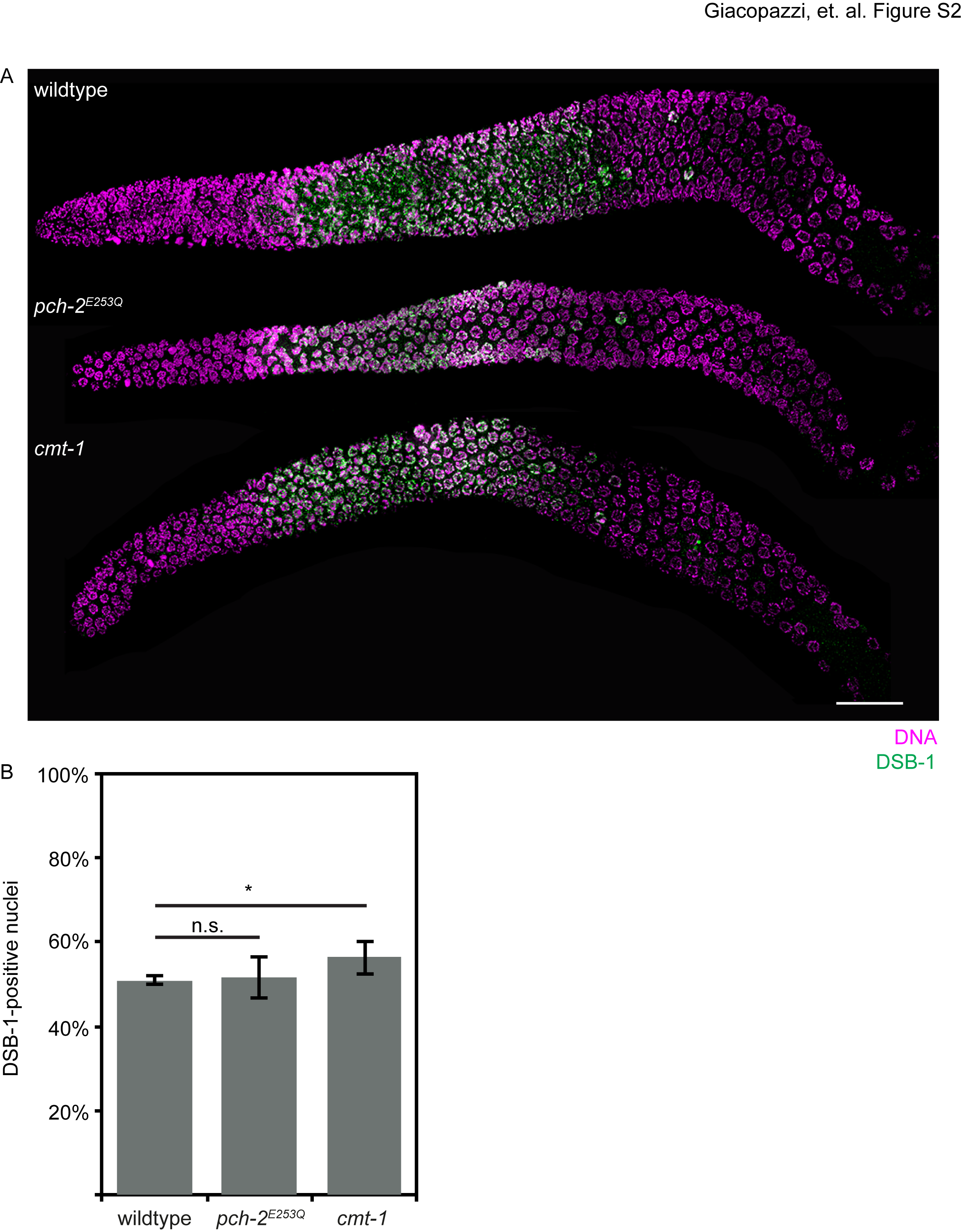

Supplement: S2 Fig — A. Whole germline images of DSB-1 and DAPI staining in a wildtype, pch-2E253Q and cmt-1 mutant germline. Scale bar indicates 20 microns. B. Quantification of percentage of DSB-1-positive nuclei in wildtype, pch-2E253Q and cmt-1 mutant germlines. Error bars indicate 95% confidence intervals. Significance was assessed by performing two-tailed t-tests. A * indicates a p value < 0.05 and an n.s. indicates not significant. (TIF) [file pgen.1008904.s002.tif]

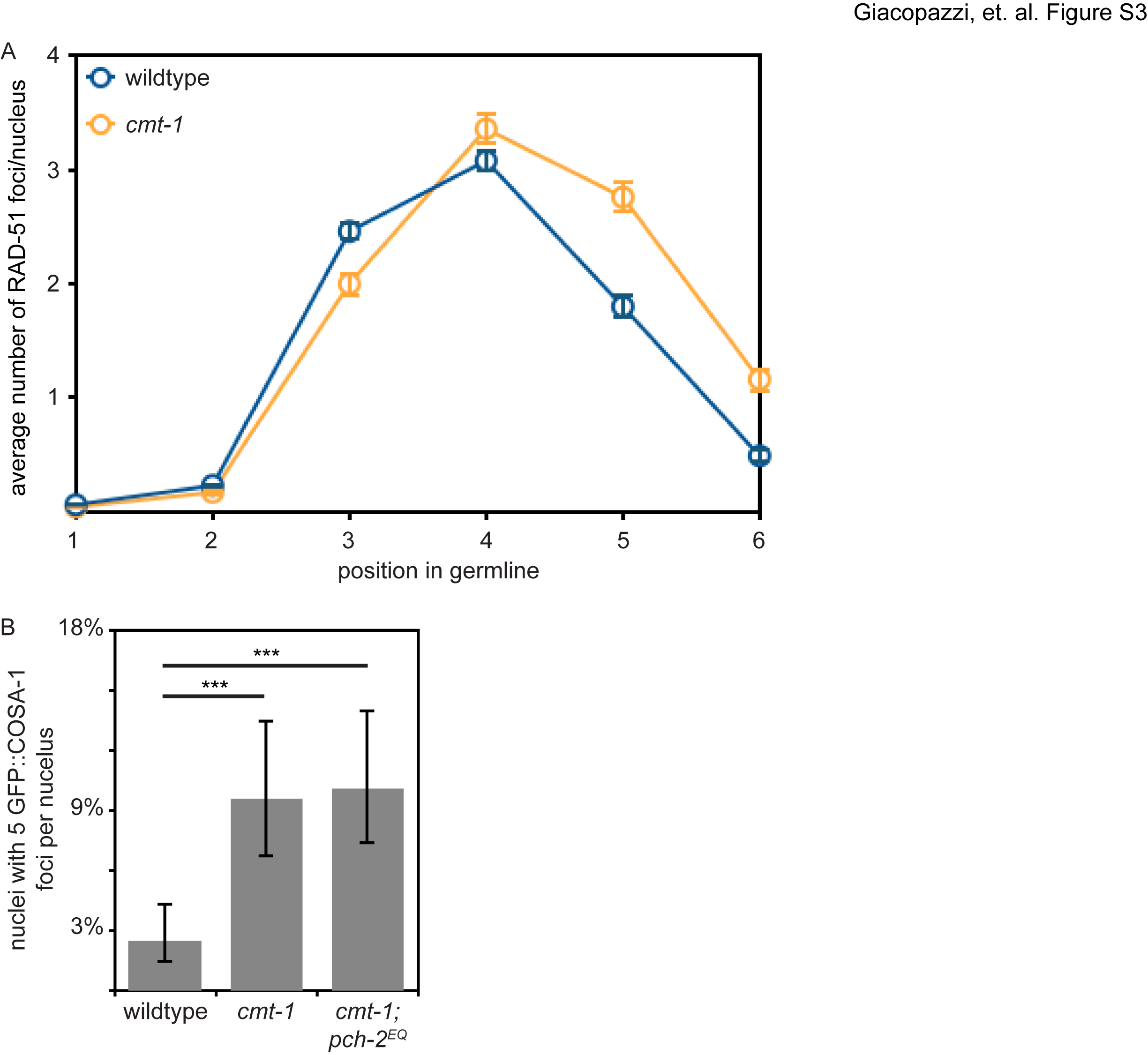

Supplement: S3 Fig — A. Timecourse of the average number of RAD-51 foci per nucleus in wildtype and cmt-1 mutant germlines. Error bars indicate 2XSEM. B. Percentage of nuclei with five GFP::COSA-1 foci in wildtype animals, cmt-1 single and cmt-1;pch-2E253Q double mutants. Error bars indicate 95% confidence intervals. Significance was assessed by performing two-tailed Fisher exact tests. A *** indicates a p value < 0.0001. (TIF) [file pgen.1008904.s003.tif]

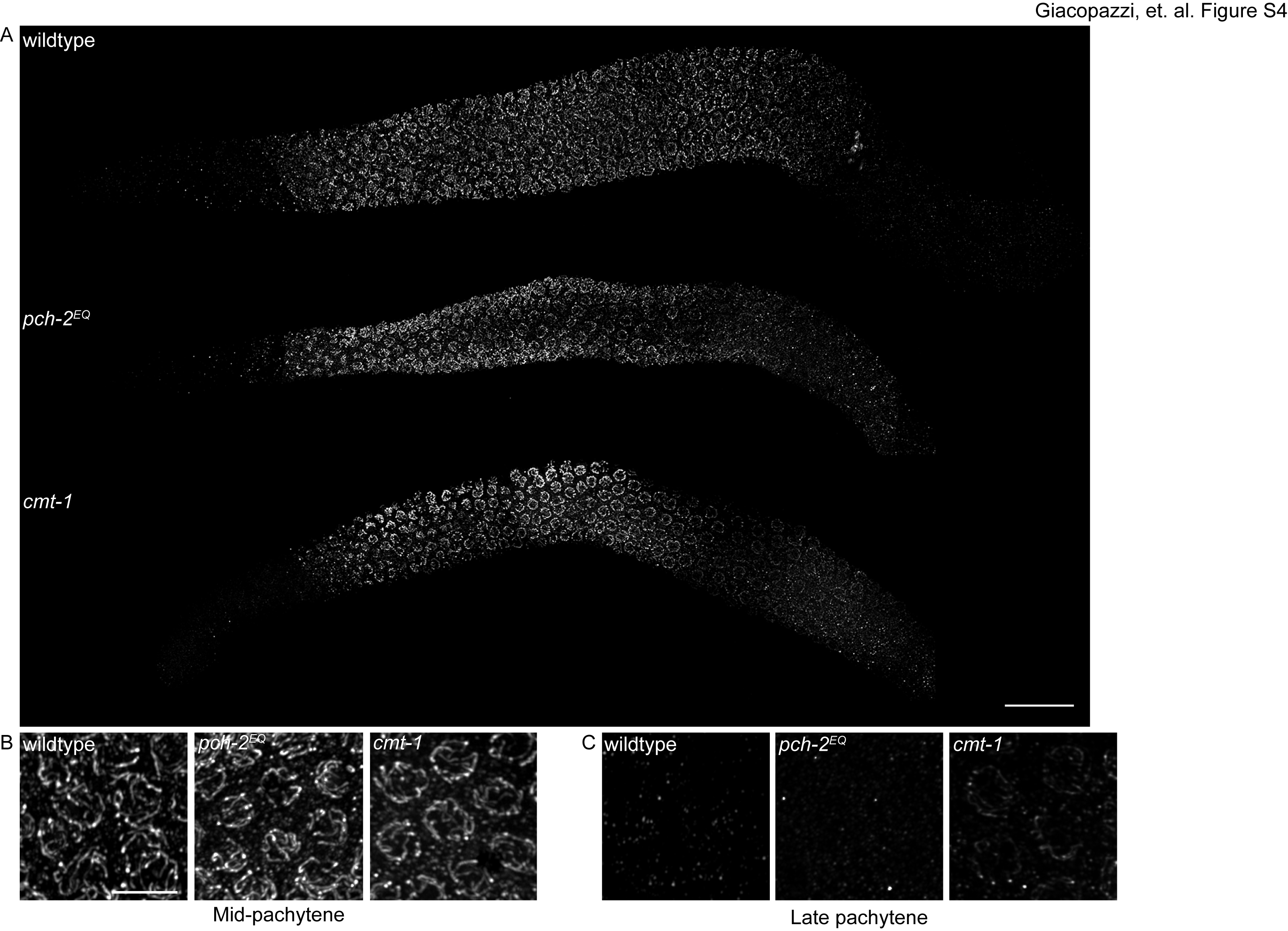

Supplement: S4 Fig — A. Whole germline images. Scale bar indicates 20 microns. B. Meiotic nuclei in mid-pachytene. C. Meiotic nuclei in late pachytene. (TIF) [file pgen.1008904.s004.tif]
