# Supplementary material for: PCH-2 collaborates with CMT-1 to proofread meiotic homolog interactions
Source: PLoS Genet. 2020 Jul 30;16(7):e1008904. doi: 10.1371/journal.pgen.1008904 (PMC7433886; doi:10.1371/journal.pgen.1008904)
Supplement: S1 Table — (DOCX) [file pgen.1008904.s005.docx]

**Table S1: Number of nuclei assayed for each genotype for all figures.**

|  | | **number of nuclei in each zone** | | | | | |
| --- | --- | --- | --- | --- | --- | --- | --- |
| **Figure** | **Genotype** | **1** | **2** | **3** | **4** | **5** | **6** |
| 2C | *syp-1* | 572 | 569 | 446 | 347 | 236 | 224 |
|  | *syp-1;pch-2Δ* | 550 | 536 | 412 | 417 | 349 | 194 |
|  | *syp-1;pch-2^EQ^* | 548 | 731 | 494 | 398 | 290 | 196 |
| 3B | wildtype | 396 | 491 | 562 | 536 | 392 | 247 |
|  | *pch-2Δ* | 473 | 530 | 537 | 485 | 375 | 210 |
|  | *pch-2^EQ^* | 417 | 605 | 528 | 446 | 409 | 295 |
| 4B | wildtype | 509 | 501 | 436 | 442 | 356 | 258 |
|  | *pch-2* | 611 | 502 | 408 | 342 | 265 | 155 |
|  | *pch-2^EQ^* | 359 | 359 | 359 | 309 | 290 | 176 |
| 6A | *syp-1* | 572 | 569 | 446 | 347 | 236 | 224 |
|  | *syp-1;cmt-1* | 396 | 554 | 494 | 494 | 426 | 256 |
| 6B | wildtype | 396 | 491 | 562 | 536 | 392 | 247 |
|  | *cmt-1* | 494 | 533 | 441 | 405 | 296 | 205 |
| S3A | wildtype | 573 | 581 | 548 | 478 | 339 | 235 |
|  | *cmt-1* | 367 | 347 | 352 | 307 | 258 | 198 |

| **Figure** | **Genotype** | **number of nuclei** |
| --- | --- | --- |
| 3D | wildtype | 854 |
|  | *pch-2Δ* | 1218 |
|  | *pch-2^EQ^* | 577 |
| 4D | wildtype | 478 |
|  | *pch-2Δ* | 412 |
|  | *pch-2^EQ^* | 237 |
| 6D | wildtype | 854 |
|  | *cmt-1* | 424 |
|  | *cmt-1;pch-2^EQ^* | 450 |
|  | *cmt-1;pch-2Δ* | 641 |
| S1B | wildtype | 301 |
|  | *pch-2^EQ^* | 259 |
|  | *cmt-1* | 214 |
| S3B | wildtype | 478 |
|  | *cmt-1* | 293 |
|  | *cmt-1; pch-2^EQ^* | 336 |
