# Supplementary material for: PCH-2 collaborates with CMT-1 to proofread meiotic homolog interactions
Source: PLoS Genet. 2020 Jul 30;16(7):e1008904. doi: 10.1371/journal.pgen.1008904 (PMC7433886; doi:10.1371/journal.pgen.1008904)
Supplement: Attachment — Submitted filename: Giacopazzi_Vong_PlosGenetics.docx [file pgen.1008904.s006.docx]

The manuscript entitled “PCH-2 collaborates with CMT-1 to proofread 1 meiotic homolog interactions”, presents work suggesting a role for PCH-2 during C. elegans meiosis that is distinct from its role in mitosis. The authors create a mutant version of PCH-2 that can bind to ATP and its substrates but cannot hydrolyze ATP. They characterize this mutant as exhibiting (low levels of) non-homologous synapsis and a loss of crossover assurance. Further, they show that a null allele of a PCH-2 adaptor protein, CMT-1, exhibits similar phenotypes. The authors suggest that these results mean that PCH-2 can bind to meiotic substrates without CMT-1 but require it to hydrolyze ATP and remodel meiotic intermediates. While these results are of sufficient interest to be published in PLOS Genetics, as the manuscript stands it needs major revisions to be acceptable. The biggest concern is the amount of data regarding non-homologous synapsis that is marked as data not shown (see below for detailed comments). Many of the authors conclusions rest on the presence or absence of non-homologous synapsis in different mutants and there is a lack of quantification or statistics. In addition to data regarding non-homologous synapsis there are a few other areas of the manuscript where the presentation and discussion of the results could be improved (see below for specifics).

Major comments:

1) As it stands, the authors present images in Fig 3C showing non-homologous synapsis for the X chromosome. In the text on line 165-166 it is stated that this also occurs on the autosomes and at a rate of 3% on the X chromosome. These data are all listed as not shown. These data need to be included and the quantification of all the chromosomes should be done.
2) The authors extrapolate their X data to all chromosomes in line 166-168 which is unnecessary if they have already done the autosome staining. Additionally, if one chromosome is non-homologously synapsed than another one should be as well in the same cell. So the 18% seems like it might be an overstatement. Have you looked at multiple PC’s in the same cells? It also seems possible that the X chromosome could behave differently than the autosomes as there is precedent for that in C. elegans.
3) The % of non-homologous synapsis should also be provided for the cmt-1 mutants since the authors say they detected similar levels (line 225 Fig 6).
4) It would be nice to have more data examining what exactly is happening with the non-homologous synapsis. By only looking at HIM-8 foci it is unclear what is happening along the entire chromosome. For example, staining with an antibody that is autosome specific (XND-1 or HTZ-1) and looking to see if the entire X chromosome is unpaired or if only part of it is. If there is a tool that marks just the X chromosome that would be even better.
5) Additionally, at the end of the results (line 255) the authors state that pch-2;cmt-1 double mutants do not display non-homologous synapsis and use this to make a claim of epistasis. Here no figure is referenced and these data are not present in any of the figures. This needs to be addressed.
6) The discussion of non-homologous synapsis and its biological relevance is lacking. No previous work on non-homologous synapsis and crossover regulation is discussed. There are examples from C. elegans, yeast and Drosophila that would flesh out the importance of these results and place them in a broader context.
7) Line 233: Address data not shown.
8) Figures 2, 3, 4, 6, and S2 are missing statistics (including what the error bars are) and p-values. Additionally, none of the figures have N values, these values should be added throughout. In the text, statements are sometimes made that are not clearly backed up with statistics. Some examples are below but the authors should confirm that their stats back up their statements:

-Line 136: “pch-2E253Q;syp-1 double mutants also initiate PC pairing in zone 2 but have significantly fewer nuclei with paired HIM-8 signals than syp-1 single mutants.” No stats are mentioned in figure legend or marked on the figure.

-Line 180: “In zone 5, we detected fewer RAD-51 foci,”- is this significant?

-Line 215: “syp-1 triple mutants, we observe wildtype levels of apoptosis, similar to cmt-1 single mutants” this looks like it might be significant. It would be nice to have the p-values and n values included for the not significant ones.

-Line 225-228: “Meiotic DNA repair in cmt-1 mutants, as visualized by the appearance and disappearance of RAD-51 (Figure S2A), ….., closely resembled that of wildtype germlines.” The repair of RAD-51 looks delayed- is that significant and, if so, biologically relevant?

9) In general composite images are hard to see and a second image in greyscale should be included. Specifically this is an issue in Fig 1 (hard to see where pch2 is), Fig 4 (it is impossible to see the RAD-51 foci), and Fig 7 (hard to see pch2 and it would be nice to have a reference image here). Also, authors might wish to consider their color choices as a lot of people struggle with the use of red and green.

Minor comments:

1) The intro is nicely written and accessible to a wider audience. However, the 2^nd^ intro paragraph could use more references regarding synapsis and pairing in c. elegans and the timing of those events in relation to crossovers.
2) In the last paper on pch-2 (Deshong et al 2014) the authors used the term quality control mechanism. I feel like that is more accurate than proofreading as it encompasses more possibilities for pch-2’s mechanism of action (whereas proofreading seems more specific). Was there are a reason for the change in terminology?

3) Line 69: This long sentence is awkward and a little hard to read.
4) Line 97: “we see a similar effect on recombination” is vague and should be changed to be clearer.
5) Is the mutant germline in Fig 1 smaller? Does this mean anything?
6) Figure 1 legend the B and C are formatted differently than the other legends.
7) Line 137-138: “ In later zones, pch-2E253Q ;syp-1 mutants more closely resemble syp-1 single mutants… **in that they are paired**.”
8) When there are fewer than 6 COSA foci, how many are there? Does it line up with one chromosome losing a CO?
9) Line 209: How was apoptosis assessed? I assume it was using the ced-1:GFP from the strain list but the authors should be clear in the methods and/or figure legend.
10) Line 278: delete the extra “localized”
