# Supplementary material for: PCH-2 collaborates with CMT-1 to proofread meiotic homolog interactions
Source: PLoS Genet. 2020 Jul 30;16(7):e1008904. doi: 10.1371/journal.pgen.1008904 (PMC7433886; doi:10.1371/journal.pgen.1008904)
Supplement: Attachment — Submitted filename: coverletter.doc [file pgen.1008904.s007.doc]

Dear Diana-

Thank you for the careful review and consideration of our manuscript. We are encouraged by the reviewers’ comments on our manuscript and appreciate their thoughtful and helpful suggestions to make this a stronger, more accessible and compelling story. As we discussed by email, before our lab was closed down in response to the COVID-19 epidemic, we were able to perform most, but not all, of the reviewer’s requested experiments to support our major conclusions. Specifically, we were able to address the points explicitly outlined in the decision letter: the quantification of non-homologous synapsis for both X chromosomes and autosomes, the inclusion of mutant and double mutant analysis, including those described as “data not shown," the addition of statistical analysis and a more detailed discussion of why we favor proofreading as PCH-2’s and CMT-1’s mechanism of action, at least in *C. elegans*. Given the recent guidance from PLoS encouraging reviewers “to carefully consider the need for additional experiments or analyses in revision requests, especially if the conclusions are adequately supported by the data,” we hope that the reviewers will appreciate the additions and changes to our manuscript. Here, we present a point-by point response. **Our responses are in bold.**

Regards,

Needhi Bhalla

Reviewer #1: In the manuscript by Giacopazzi, Vong et al, the authors characterize the in vivo phenotypes of a mutant in PCH-2 (pch-2E253Q). In vitro characterization previously showed that this mutant could bind its substrate but could not hydrolyze ATP. They find that pch-2E253Q mutants localize PCH-2E253Q but have a delay in pairing, accelerate synapsis, have a small fraction of non-homologous synapsis, and lose crossover assurance. The phenotype of non-homologous synapsis was different in the pch-2E253Q than in the pch-2 mutants, suggesting that the pch-2E253Q mutant is trapping an intermediate. They then ask if CMT-1 is needed for these events. They find that the phenotypes of cmt-1 mutants are similar to that of pch-2E253Q mutants, with a delay in pairing, accelerated synapsis, non-homologous synapsis, and reduced crossover assurance.

This manuscript details interesting results to test their hypothesis that they can reveal intermediates with the pch-2E253Q mutants and that the adaptor CMT-1 is not required for binding substrates but is required to remodel them. However, there are some further questions that need to be addressed:

1) In Figure 2C, the authors are arguing that they can trap the pairing intermediates in pch-2E253Q mutants. It would be more convincing if they directly show the difference between pch-2delta and pch-2E253Q, along with wildtype.

**This data is now included in Figure 2C.**

2) Similarly, the comparison of HIM-8 foci in pch-2E253Q and pch-2delta is needed to show that this is a defect specific to trapping intermediates.

**This data is now included in Figure 3B and quantified in Figure 3C.**

3) What is the range of numbers and percent of each of GFP::COSA-1 foci? Having this range will help understand the severity of the defect.

**We clarified this in the text and figures: we are specifically assaying the percentage of nuclei with 5 GFP::COSA-1 foci per nucleus. We did not observe nuclei with less than five GFP::COSA-1 foci per nucleus.**

4) Is there an increase in chromosome mis-segregation in the pch-2E253Q mutants, or less viable offspring?

**No, this data is now included in Table 1.**

5) Does the role of CMT-1 in activating apoptosis require PCH-2 and ATP hydrolysis? In the pch-2E253Q mutant, what are the levels of apoptosis and does the mutant decrease apoptosis in syp-1 mutants, like cmt-1 mutants do?

**These data are now included in Figure 5C.**

6) To be sure that CMT-1 and PCH-2 are acting together to disrupt non-homologous synapsis, the cmt-1; pch-2E253Q double mutant experiment should be performed to see if there is an additive effect or not.

**This data is now included in Figure 6D.**

7) I am struggling with the role of PCH-2 in proofreading versus a cell-cycle regulator needed to couple pairing and synapsis. I am wondering if the authors can discuss these differences further and focus on why they are suggesting proofreading?

**We discuss why we favor the model of proofreading, versus misregulation of meiotic progression, to explain our results in our Discussion:**

**“We previously showed that loss of PCH-2 accelerated homolog pairing, synapsis and recombination, coordinating these meiotic prophase events [50]. Here, we argue that PCH-2 acts directly on chromosomes to proofread meiotic interhomolog interactions and ensure their fidelity. This role is supported by several pieces of data. First, PCH-2 localizes to meiotic chromosomes, both before and after synapsis [50]. This localization is functional, as demonstrated by its persistence in the synaptonemal complex and correlation with an extension of homolog access in mutants defective in recombination [50, 70]. Second, despite the defects we report, *pch-2Δ*, *pch-2^E253Q^* and *cmt-1* mutants do not show acceleration of meiotic progression, as assayed by SUN-1 phosphorylation [50], appearance of transition zone nuclei (Figures 1, 7 and [50]), ZIM-2 (Figure S1) or DSB-1 localization (Figure S2), indicating that PCH-2 and CMT-1 do not coordinate these meiotic prophase events indirectly through misregulation of meiotic progression. Finally, meiotic defects associated with loss of CMT-1 rely on PCH-2 function (Figure 6D), unlike what we observe in mitosis [66]. In mitosis, CMT-1 is epistatic to PCH-2, which we attribute to misregulation of soluble pools of Mad2. Soluble pools of at least one meiotic HORMAD, HTP-1, have been implicated in ensuring both accurate homolog synapsis and proper meiotic progression [23]. If PCH-2 and CMT-1 were regulating homolog pairing, synapsis and recombination via control of meiotic progression, we might predict that CMT-1 would be epistatic to PCH-2 in meiosis, as it is in mitosis. Taken together, these data favor a model in which PCH-2 and CMT-1 proofread interactions between homologous chromosomes that underlie pairing, synapsis and recombination in *C. elegans*. Since pairing, synapsis and recombination depend on meiotic HORMADs adopting their closed conformations [45], we hypothesize that these interactions between homologous chromosomes either involve or are stabilized by closed versions of meiotic HORMADs and PCH-2, in collaboration with CMT-1, remodels them to disassemble or destabilize them. In systems such as plants, yeast and mice, we propose that PCH-2 plays a similar role in addition to removing or relocalizing meiotic HORMADs to signal meiotic progression [28, 30-32].”**

Minor issues:
1) When referring to yeast proteins and genes, the yeast terminology should be used. Pch2 = protein, PCH2 = gene

**We have corrected this.**

2) In line 229-231, the authors conclude that PCH-2 can bind the meiotic substrates in the absence of CMT-1, but they do not show that until later. I found this statement confusing.

**We have attempted to clarify this: “The meiotic phenotypes of *cmt-1* mutants are more similar to that of *pch-2^E253Q^* mutants than to that of *pch-2Δ* mutants, suggesting that PCH-2 can bind its meiotic substrates effectively in the absence of CMT-1 but CMT-1 is required for PCH-2’s hydrolysis of ATP.”**

3) Line 149 get rid of comma after “in contrast”

**We have corrected this.**

4) Line 277-278 unneeded “localized”.

**We have removed this.**

Reviewer #2:

The manuscript entitled “PCH-2 collaborates with CMT-1 to proofread 1 meiotic homolog interactions”, presents work suggesting a role for PCH-2 during C. elegans meiosis that is distinct from its role in mitosis. The authors create a mutant version of PCH-2 that can bind to ATP and its substrates but cannot hydrolyze ATP. They characterize this mutant as exhibiting (low levels of) non-homologous synapsis and a loss of crossover assurance. Further, they show that a null allele of a PCH-2 adaptor protein, CMT-1, exhibits similar phenotypes. The authors suggest that these results mean that PCH-2 can bind to meiotic substrates without CMT-1 but require it to hydrolyze ATP and remodel meiotic intermediates. While these results are of sufficient interest to be published in PLOS Genetics, as the manuscript stands it needs major revisions to be acceptable. The biggest concern is the amount of data regarding non-homologous synapsis that is marked as data not shown (see below for detailed comments). Many of the authors conclusions rest on the presence or absence of non-homologous synapsis in different mutants and there is a lack of quantification or statistics. In addition to data regarding non-homologous synapsis there are a few other areas of the manuscript where the presentation and discussion of the results could be improved (see below for specifics).

Major comments:

1) As it stands, the authors present images in Fig 3C showing non-homologous synapsis for the X chromosome. In the text on line 165-166 it is stated that this also occurs on the autosomes and at a rate of 3% on the X chromosome. These data are all listed as not shown. These data need to be included and the quantification of all the chromosomes should be done.

**We have included the quantification for the X chromosome and Chromosome V in Figures 3C, 3D, S1A and S1B.**

2) The authors extrapolate their X data to all chromosomes in line 166-168 which is unnecessary if they have already done the autosome staining. Additionally, if one chromosome is non-homologously synapsed than another one should be as well in the same cell. So the 18% seems like it might be an overstatement. Have you looked at multiple PC’s in the same cells? It also seems possible that the X chromosome could behave differently than the autosomes as there is precedent for that in C. elegans.

**The reviewer is correct that individual nuclei may have multiple non-homologously synapsed chromosomes and that 18% may be an overstatement. We have included this caveat in our manuscript. We have not looked at multiple PCs in the same cells since this experiment is technically challenging: To identify nuclei with completely synapsed chromosomes and unpaired PCs uses all three fluorophores that we can image on our microscope. Including an additional antibody against another chromosome’s PCs is not possible.**

3) The % of non-homologous synapsis should also be provided for the cmt-1 mutants since the authors say they detected similar levels (line 225 Fig 6).

**This data is included in Figures 6D, S1A and S1B.**

4) It would be nice to have more data examining what exactly is happening with the non-homologous synapsis. By only looking at HIM-8 foci it is unclear what is happening along the entire chromosome. For example, staining with an antibody that is autosome specific (XND-1 or HTZ-1) and looking to see if the entire X chromosome is unpaired or if only part of it is. If there is a tool that marks just the X chromosome that would be even better.

**We were not able to perform this experiment before our lab was shut down. However, our inability to perform this experiment does not substantially affect our conclusions.**

5) Additionally, at the end of the results (line 255) the authors state that pch-2;cmt-1 double mutants do not display non-homologous synapsis and use this to make a claim of epistasis. Here no figure is referenced and these data are not present in any of the figures. This needs to be addressed.

**This data is included in Figures 6C and 6D.**

6) The discussion of non-homologous synapsis and its biological relevance is lacking. No previous work on non-homologous synapsis and crossover regulation is discussed. There are examples from C. elegans, yeast and Drosophila that would flesh out the importance of these results and place them in a broader context.

**We have included this in the introduction:**

**“Coordinating the events of pairing, synapsis and crossover recombination is essential for their proper progression. For example, defects that uncouple pairing and synapsis can produce situations in which non-homologous chromosomes are inappropriately synapsed and unable to undergo crossover recombination. Mutations that produce non-homologous synapsis often identify mechanisms that are important for homolog pairing. For example, in maize, budding yeast and mice, where the initiation of meiotic recombination plays an integral role in promoting accurate homolog pairing and synapsis, mutations in genes involved in homologous recombination can produce non-homologous synapsis to varying degrees [9-17]. In *Drosophila* and *C. elegans*, two organisms in which correct pairing and synapsis can occur independent of recombination events [18, 19], other mechanisms ensure correct homologous synapsis, including integrity of meiotic chromosome axes [20-23], highly regulated chromosome movement [24, 25], centromere clustering [26] and specific histone modifications [27].”**

7) Line 233: Address data not shown.

**These data are now included in Figures 6D and S3B.**

8) Figures 2, 3, 4, 6, and S2 are missing statistics (including what the error bars are) and p-values. Additionally, none of the figures have N values, these values should be added throughout. In the text, statements are sometimes made that are not clearly backed up with statistics. Some examples are below but the authors should confirm that their stats back up their statements:

**We sincerely apologize for this lapse. We have indicated what error bars signify in all figures and included statistical analysis either in the text or the Figures and Figure Legends. In addition, we have included a table (Table S1) that lists the number of nuclei scored for each experiment. We have also specifically addressed the questions the reviewer raised.**

-Line 136: “pch-2E253Q;syp-1 double mutants also initiate PC pairing in zone 2 but have significantly fewer nuclei with paired HIM-8 signals than syp-1 single mutants.” No stats are mentioned in figure legend or marked on the figure.

**This statistic is now included in the text.**

-Line 180: “In zone 5, we detected fewer RAD-51 foci,”- is this significant?

**Yes, and this statistic is now included in the text.**

-Line 215: “syp-1 triple mutants, we observe wildtype levels of apoptosis, similar to cmt-1 single mutants” this looks like it might be significant. It would be nice to have the p-values and n values included for the not significant ones.

**The difference between *cmt-1* single mutants and *syp-1;spo-11;cmt-1* triple mutants is significant, (p value < 0.05) and this statistic is included in the text. However, this is common in our experiments and not necessarily biologically relevant, which is why we focus on the difference between *syp-1;spo-11* double mutants and *syp-1;spo-11;cmt-1* triple mutants in the Figure. We have included the p values for relevant not significant findings.**

-Line 225-228: “Meiotic DNA repair in cmt-1 mutants, as visualized by the appearance and disappearance of RAD-51 (Figure S2A), ….., closely resembled that of wildtype germlines.” The repair of RAD-51 looks delayed- is that significant and, if so, biologically relevant?

**We have included this observation: However, we did observe a delay in RAD-51 removal in *cmt-1* mutants (zones 5 and 6, p value > 0.0001, two-tailed Fisher’s exact test). It is unclear whether this delay is biologically relevant, since we only observe a slight delay in DSB-1 removal (Figure S2B).”**

9) In general composite images are hard to see and a second image in greyscale should be included. Specifically this is an issue in Fig 1 (hard to see where pch2 is), Fig 4 (it is impossible to see the RAD-51 foci), and Fig 7 (hard to see pch2 and it would be nice to have a reference image here). Also, authors might wish to consider their color choices as a lot of people struggle with the use of red and green.

**These images are now included as greyscale images, either in the Figure itself (Figure 4) or as a supplementary Figure (Figure S4). We have also changed the color choice to magenta and green in situations where we previously used red and green.**

Minor comments:

1) The intro is nicely written and accessible to a wider audience. However, the 2^nd^ intro paragraph could use more references regarding synapsis and pairing in c. elegans and the timing of those events in relation to crossovers.

**We have included additional references in this paragraph. We mention the relationship between pairing, synapsis and recombination in *C. elegans* in the following paragraph.**

2) In the last paper on pch-2 (Deshong et al 2014) the authors used the term quality control mechanism. I feel like that is more accurate than proofreading as it encompasses more possibilities for pch-2’s mechanism of action (whereas proofreading seems more specific). Was there are a reason for the change in terminology?

**We discuss why we favor the model of proofreading, versus misregulation of meiotic progression, to explain our results in our Discussion.**

**“We previously showed that loss of PCH-2 accelerated homolog pairing, synapsis and recombination, coordinating these meiotic prophase events [50]. Here, we argue that PCH-2 acts directly on chromosomes to proofread meiotic interhomolog interactions and ensure their fidelity. This role is supported by several pieces of data. First, PCH-2 localizes to meiotic chromosomes, both before and after synapsis [50]. This localization is functional, as demonstrated by its persistence in the synaptonemal complex and correlation with an extension of homolog access in mutants defective in recombination [50, 70]. Second, despite the defects we report, *pch-2Δ*, *pch-2^E253Q^* and *cmt-1* mutants do not show acceleration of meiotic progression, as assayed by SUN-1 phosphorylation [50], appearance of transition zone nuclei (Figures 1, 7 and [50]), ZIM-2 (Figure S1) or DSB-1 localization (Figure S2), indicating that PCH-2 and CMT-1 do not coordinate these meiotic prophase events indirectly through misregulation of meiotic progression. Finally, meiotic defects associated with loss of CMT-1 rely on PCH-2 function (Figure 6D), unlike what we observe in mitosis [66]. In mitosis, CMT-1 is epistatic to PCH-2, which we attribute to misregulation of soluble pools of Mad2. Soluble pools of at least one meiotic HORMAD, HTP-1, have been implicated in ensuring both accurate homolog synapsis and proper meiotic progression [23]. If PCH-2 and CMT-1 were regulating homolog pairing, synapsis and recombination via control of meiotic progression, we might predict that CMT-1 would be epistatic to PCH-2 in meiosis, as it is in mitosis. Taken together, these data favor a model in which PCH-2 and CMT-1 proofread interactions between homologous chromosomes that underlie pairing, synapsis and recombination in *C. elegans*. Since pairing, synapsis and recombination depend on meiotic HORMADs adopting their closed conformations [45], we hypothesize that these interactions between homologous chromosomes either involve or are stabilized by closed versions of meiotic HORMADs and PCH-2, in collaboration with CMT-1, remodels them to disassemble or destabilize them. In systems such as plants, yeast and mice, we propose that PCH-2 plays a similar role in addition to removing or relocalizing meiotic HORMADs to signal meiotic progression [28, 30-32].”**

3) Line 69: This long sentence is awkward and a little hard to read.

**We clarified this: “Meiotic HORMADs appear to adopt two conformations: a “closed” version, that forms upon binding a short peptide within other proteins [45]; and a more extended, or “unlocked,” version [46].”**

4) Line 97: “we see a similar effect on recombination” is vague and should be changed to be clearer.

**We have removed this sentence.**

5) Is the mutant germline in Fig 1 smaller? Does this mean anything?

**There is variability in germline size, even among wildtype germlines. We have not noticed a consistent phenotype of smaller germlines in *pch-2^E253Q^* mutants.**

6) Figure 1 legend the B and C are formatted differently than the other legends.

**Yes, it seemed easier than repeating the same information.**

7) Line 137-138: “ In later zones, pch-2E253Q ;syp-1 mutants more closely resemble syp-1 single mutants… **in that they are paired**.”

**We corrected this.**

8) When there are fewer than 6 COSA foci, how many are there? Does it line up with one chromosome losing a CO?

**Yes, there are 5 GFP::COSA-1 foci when there are fewer than six. We have made this more clear in the manuscript.**

9) Line 209: How was apoptosis assessed? I assume it was using the ced-1:GFP from the strain list but the authors should be clear in the methods and/or figure legend.

**The methods now include this sentence: “Scoring of germline apoptosis was performed as previously described in [43] in strains containing *bcIs39 [lim-7p::ced-1::GFP + lin-15(+)]* with the following exceptions.”**

10) Line 278: delete the extra “localized”

**We have corrected this.**

Reviewer #3: The manuscript by Giacopazzi et al., provides insight into the role of the enigmatic AAA-ATPase PCH-2 in ensuring faithful meiotic prophase events. Taking advantage of a mutation in the conserved Walker B motif that is able to bind ATP but no hydrolyze it, the authors monitor localization, pairing, synapsis, and meiotic recombination progression. They find that pch-2E235Q has delayed pairing (of the X chromosome), faster SC formation leading to some nonhomologous synapsis, and relatively normal RAD-51 kinetics, but a slight reduction in the frequency of COSA-1 foci, suggesting a problem with crossover assurance. Most of these phenotypes are also observed in the p31comet ortholog, CMT-1; however, unlike its role in the spindle assembly checkpoint, CMT-1 is not required to localize PCH-2 to meiotic chromosomes. Together these results suggest that CMT-1 plays a distinct role in monitoring meiotic prophase events versus its canonical role in the spindle assembly checkpoint pathway. There are some interesting data presented, although not much insight into the underlying mechanism. In many figures, there are no statistics. Further, including the pch-2 null mutant in the analysis and reorganizing the figures would make it easier to compare pch-2 null, pch-2E235Q and cmt-1.

Abstract: “In vitro, this mutant binds its substrates . . .” – to my knowledge this has only been demonstrated for MAD-2 in the spindle assembly checkpoint and not for potential meiotic substrates (Ye, et al., 2015). This should be changed to reflect this. Further on line 29 in the abstract, please change “indicate” to “suggest” as no direct binding of meiotic substrates is shown in this manuscript.

**We have changed this to: “*In vitro*, this mutant can bind a known substrate but is unable to remodel it.” We have also changed “indicate” to “suggest” in line 29.**

I recommend that the data for the cmt-1 mutant be incorporated into the figures for the pch-2E235Q as indicated below.

**We appreciate this recommendation but feel that it makes more sense in presenting the narrative to have separate figures for *pch-2^E253Q^* mutants and *cmt-1* mutants, particularly if we will include *pch-2* null mutants as an additional control in our graphs that include *pch-2^E253Q^* mutants. In addition, some experiments were performed by different authors and therefore have different controls, such as Figure S3A.**

Figure 1: Please indicate that this is antibody staining. There is significantly more background/non-nuclear staining in the mutant compared to wild type. Is this consistent over different experiments? Either replace the figure or suggest why it appears different. Please add the PCH-2 staining in cmt-1 mutant to this figure instead of figure 7.

**The Figure legend states: “Meiotic nuclei in mid-pachytene (B) and late pachytene (C) stained with DAPI and antibodies against PCH-2 in wildtype animals and *pch-2^E253Q^* mutants.” The background staining is not consistent over many experiments but we were unable to perform this experiment again before our lab was shut down. All three germlines (wildtype, *pch-2^E253Q^* and *cmt-1*) are now shown together in Figure S4.**

Figure 2: As the X chromosome has different properties compared to the autosomes, it would be nice to include analysis of pairing of an autosome. Please also add the analysis of pairing of the pch-2 null and cmt-1 mutants in this figure (Fig 6A). Statistics should also be included.

**We were not able to perform the analysis of pairing of an autosome before our lab was shutdown. However, the implication from our analysis of non-homologous synapsis with ZIM-2 indicates that similar dynamics would be observed. Thus, our inability to perform this experiment does not substantially affect our conclusions. We have included the analysis of *pch-2* null mutants and the appropriate statistical analysis.**

Figure 3: Please quantify the extent of non-homologous synapsis. The data for ZIM-1 and ZIM-2 should be included, as well as the data for pch-2 null and cmt-1 mutants (Figure 6B).

**This data for HIM-8 and ZIM-2 is quantified in Figures 3D, 6D, and S1B.**

Figure 4: Please add the pch-2 null and cmt-1 data from Supplemental Figure 2 here as well as statistics.

**We have included the *pch-2* null mutant data and the appropriate statistical analysis.**

Minor: Several times throughout the manuscript meiotic DNA repair is written (line 98, line172, and others), I think is should be DSB. There is also a typo on line 278.

**While the appearance of RAD-51 reports on the progress of double strand break formation, its disappearance reports on entry into a DNA repair pathway. Therefore, following the kinetics of RAD-51 appearance and disappearance reports on the progress of DNA repair. Further, we previously showed that loss of PCH-2 had no effect on double strand break formation. We have attempted to make this more clear in the manuscript. We corrected the typo.**
